# Precision Photochemistry: Every Photon Counts

**DOI:** 10.1002/anie.202502651

**Published:** 2025-08-04

**Authors:** Fred Pashley‐Johnson, Xingyu Wu, Joshua A. Carroll, Sarah L. Walden, Hendrik Frisch, Andreas‐Neil Unterreiner, Filip E. Du Prez, Hans‐Achim Wagenknecht, Javier Read de Alaniz, Ben L. Feringa, Alexander Heckel, Christopher Barner‐Kowollik

**Affiliations:** ^1^ School of Chemistry and Physics Centre for Materials Science Queensland University of Technology (QUT) 2 George Street Brisbane Queensland 4000 Australia; ^2^ Polymer Chemistry Research Group, Centre of Macromolecular Chemistry, Department of Organic and Macromolecular Chemistry Ghent University Krijgslaan 281‐S4 Ghent 9000 Belgium; ^3^ School of Environment and Science Griffith University 170 Kessels Road Nathan Queensland 4111 Australia; ^4^ Institute of Physical Chemistry Karlsruhe Institute of Technology (KIT) Kaiserstraße 12 76131 Karlsruhe Germany; ^5^ Institute of Organic Chemistry Karlsruhe Institute of Technology (KIT) Fritz‐Haber‐Weg 6 76131 Karlsruhe Germany; ^6^ Department of Chemistry and Biochemistry University of California Santa Barbara California 93106 USA; ^7^ Stratingh Institute for Chemistry University of Groningen Nijenborgh 3 Groningen 9747 AG The Netherlands; ^8^ Institute for Organic Chemistry and Chemical Biology Goethe University Frankfurt Max‐von‐Laue‐Straße 9 60438 Frankfurt (Main) Germany; ^9^ Institute of Nanotechnology (INT) and Institute of Functional Interfaces (IFG) Karlsruhe Institute of Technology (KIT) Hermann‐von‐Helmholtz‐Platz 1 76344 Eggenstein‐Leopoldshafen Germany

**Keywords:** Action plot, Light source, Photochemistry, Quantum yield, Wavelength

## Abstract

Photochemistry is undergoing a precision transformation. Through technological advancements, such as the advent of light emitting diodes and monochromatic lasers, chemists are now able to use photons not only as an energy source but also as a tool for directing photochemical processes with both wavelength and spatiotemporal precision. Enabled by these technologies, the discovery that photochemical action often does not align with molar extinction has catalysed the growth of the research field that we coin *Precision Photochemistry*. We explain how precision photochemistry stands on four fundamental pillars: molar extinction, wavelength‐dependent quantum yield, concentration of the chromophores, and the length of the irradiation. Each of these four pillars are intrinsically linked and dictate the experimental conditions that should be used (e.g., wavelength, light intensity, and solvent system), as we demonstrate through simulations of a photochemical uncaging system. Building on these pillars, we propose a concrete definition for *Precision Photochemistry* and highlight important fields within chemistry that will benefit from careful consideration of them. Finally, we address key experimental considerations that must be taken into account when conducting precision photochemistry including the light source, the reaction setup, and the method for determining (wavelength‐dependent) quantum yields. These factors are critical in furthering the development of the field of *Precision Photochemistry*.

## Graphical abstract

Every photon used in a photochemical reaction has the power to dictate the outcome of the reaction. We present a framework whereby understanding the role that each photon has in a reaction can enablechemists to realise the true power of photochemistry and dramatically improve reaction outcomes.We coin this field Precision Photochemistry.

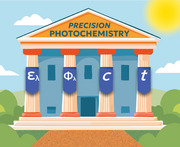

## 1 The Four Pillars of Precision Photochemistry

In 1819, Theodor von Grotthuss postulated that

*“Light strives to pursue as easily as possible its path in the substance in which it penetrates, and this tendency has to be the larger, the less similar is its colour to the natural colour of the substance”*.^[^ ^1^, ^2^ ^]^

In other words, he proposed that photochemical action is greater in systems that absorb the colour of light incident upon them. Subsequently, Grotthuss and Draper independently established the first law of photochemistry: “Only light which is absorbed by a system can cause chemical change”.^[^ ^2^ ^]^ Later, the development of the Beer–Bouguer–Lambert law built upon their work by mathematically linking the absorbance (or optical density) of a molecule (*A*) to its concentration (*c*), its molar extinction coefficient (*ε*_λ_) and the path length of the light in the medium (*l*).^[^ ^3^ ^]^ Thus, a molecule that is irradiated with light that falls within its absorption maximum will undergo more absorption events than if irradiated with light of a different wavelength. For centuries, this has meant that photochemists have irradiated their samples with a wavelength that matches the maximum absorption of the system (*λ*_max_) in order to induce the maximum photochemical change.

A lesser‐known fact in photochemistry, however, is that not all absorption events are equal. In 2017, the groups of Barner–Kowollik and Gescheidt showed that the photoreactivity of an oxime ester photoinitiator was enhanced when irradiated with light that was red‐shifted relative to the *λ*_max_ of the system.^[^ ^4^ ^]^ This finding led to the development of the modern‐day photochemical action plot methodology that has since revealed similarly red‐shifted photoreactivity in many systems.^[^ ^5^, ^6^, ^7^ ^]^ The observed red‐shifting of photochemical reactivity compared to the extinction spectrum evidences that not only *ε*_λ_ is important for achieving high photochemical reaction yields, but also the wavelength‐dependent reaction quantum yield (i.e., the number of reactions that occur per photon absorbed) of the system (*Φ_λ_*). By exploiting the co‐dependence of *ε*_λ_ and *Φ*_λ_ when selecting a wavelength for a photochemical process, many orthogonal,^[^ ^8^, ^9^ ^]^ cooperative,^[^ ^10^ ^]^ synergistic,^[^ ^11^ ^]^ and antagonistic^[^ ^12^, ^13^, ^14^ ^]^ photochemical systems have been developed.

Of equal importance to the quantum yield (*Φ*_λ_) and *ε*_λ_ is the duration of the irradiation (*t*) or, more relevant, the number of photons incident upon a sample over a given amount of time (photon flux). For the majority of complex molecules, it is known that when there is some energy in the system, there is a non‐zero chance that an excited vibrational state of the ground state will be populated, which can allow excitation when irradiated with any wavelength of light. In other words, the absorption of a molecule is never zero due to the Boltzmann population of the excited vibrational states. By irradiating for a specific amount of time–including, in theory, infinitely long times–almost all photochemical reactions can be brought to full conversion at any wavelength provided there is no competing reaction. In fact, there always exists a non‐zero probability of an absorption event that leads to a reaction event yielding a given photoproduct.^[^ ^15^, ^16^ ^]^

In addition to time, the concentration (*c*) of the photoreactive chromophores is critical. In 2019 and 2023, Heckel and coworkers outlined the mathematical framework that highlights the importance of concentration in the context of λ‐orthogonal uncaging reactions.^[^ ^17^, ^18^ ^]^ They showed that not only is the initial concentration of the photoreactive components vital to the orthogonality of a photoreaction, but also the time‐dependence of the concentration throughout the duration of the irradiation.

**Figure 1:**
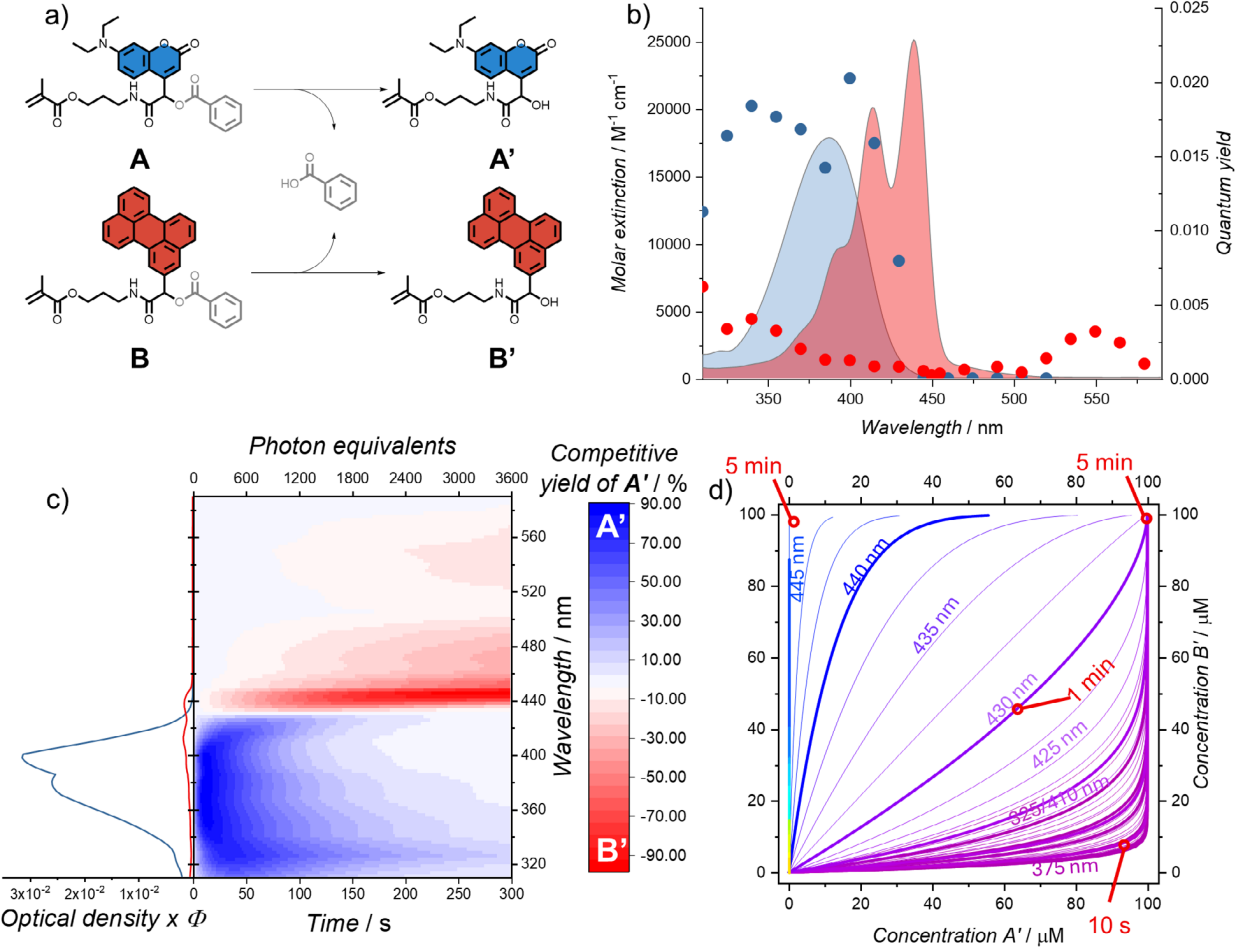
a) Chemical scheme of the photocleavage reaction that takes place when the Passerini adducts (A and B) undergo photouncaging to yield A’ and B’, with the loss of benzoic acid; (b) Molar extinction spectra of A (blue line) and B (red line) and wavelength‐dependent quantum yields for the photochemical cleavage of A (blue dots) and B (red dots) – data adapted from reference^[^ ^15^ ^]^; (c) left side: plot of the product of optical density and the wavelength dependent quantum yield of each of the starting materials in an equimolar mixture at *t* = 0; right side: the time‐dependent competitive yield of a mixture of 100 µM A and B at different wavelengths. Blue shows a higher competitive yield of A’, red shows a higher yield of B’ and white shows that there is no selectivity at that wavelength. Each sample was irradiated with a computed, monoaromatic light source with a photon flux of 1.2 µmol s^−1^ (V = 1 mL); (d) Reaction trajectories at different wavelengths extracted from the simulations in panel (c). The trajectories are coloured using the colour that corresponds to the wavelength in the electromagnetic spectrum. The first wavelength is 315 nm and the last is 590 nm with a 2.5 nm step between each simulation. 10 nm intervals are highlighted in bold.

Thus, precision photochemistry stands on four pillars: *ε*_λ_, *Φ*_λ_, *t*, and *c*. To illustrate the interactive nature of these four parameters, we have exploited the mathematical framework developed by Heckel and colleagues (that is briefly outlined in the Supplementary Information, and discussed in detail in reference^[^ ^18^ ^]^) to analyse a system that we published previously on a set of wavelength‐orthogonal photo‐uncaging molecules (Figure 1a).^[^ ^15^ ^]^ The wavelength dependence of the first two pillars, *ε*_λ_ and *Φ*_λ_, is illustrated in Figure 1b. Both reactions (the photo‐uncaging of **A** and **B**, shown in Figure 1a) display a strong mismatch between their extinction spectra and their reactivity spectra (*Φ*_λ_), as has been observed in many systems before. A combination of physical and chemical factors may all affect the outcome of photochemical reactions: for example, the impact of photochemical reactions occurring from different excited states, photostationary states governing wavelength‐dependent reactivity, and the effect of ground‐state aggregation have all been presented by Turro and colleagues previously.^[^ ^19^ ^]^ Specifically addressing the often observed mismatch between absorbance and wavelength‐resolved photochemical reactivity, the Barner–Kowollik team has presented theories on the topic,^[^ ^5^, ^6^, ^20^ ^]^ including an experimentally‐backed theory for a key mechanism that underpins the often red‐shifted reactivity.^[^ ^21^ ^]^

Upon initial inspection of Figure 1b, it is not immediately obvious that a wavelength regime exists where **A** and **B** can be selectively cleaved in a sequence‐independent manner. Here, the importance of the final two pillars – *t* and *c* – comes into play. As can be seen on the left side of Figure 1c, guidance on which chromophore will preferentially react at the beginning of a reaction can be extracted by interrogating the product of optical density (OD, a concentration‐ and geometry‐dependent representation of *ε*_λ_) and the wavelength‐dependent quantum yield (*Φ*_λ_). The plot on the left of Figure 1c can be read as a measure of how likely a photon is to trigger each reaction to occur at that wavelength for, in this case, an equimolar mixture of the two chemicals, **A** and **B**. A concentration change of any of the two species would be reflected as a change in the optical density. The plot encompasses the first three pillars of photochemistry *ε*_λ_, *Φ*_λ_, and *c*, and demonstrates that at the beginning of the irradiation (*t_0_*) the cleavage of **A** is preferred for *λ* < 430 nm, and the cleavage of **B** is preferred for *λ* > 430 nm. However, the intricate interplay between the four pillars is dynamic in time. As one of the substances is consumed, the optical density changes accordingly.

For the following discussion, we apply the aforementioned mathematical framework that has been used to simulate the wavelength‐dependent behaviour.^[^ ^17^, ^18^ ^]^ It is important to note that the experimental quantum yield data has a sampling interval of 15 nm that has been interpolated linearly. Interpolation of low‐resolution data has the potential to overlook quantum yield maxima that appear with small variations in irradiation wavelength. Distinct local maxima in wavelength‐dependent reactivity have been reported in several cases with only minor change in the irradiation wavelength.^[^ ^7^, ^15^, ^20^ ^]^ Ideally, the recording of *Φ*_λ_ would be conducted automatically, and with 1 nm intervals, as is commonly done for extinction spectra. However, due to the lack of automation and large experimental effort required to record *Φ*_λ_ values, this is currently not possible. In future work, an increase in this sampling interval through automation of the photochemical action plot procedure will be key in achieving maximum precision.^[^ ^15^ ^]^

At first, the question arises of how to quantify the selectivity of our two exemplary reactions. A possible approach is to define a *product excess* (%pe) in analogy to the enantiomeric excess (%ee) in a kinetically resolved stereochemical reaction. Therefore, %pe would be highest just after *t_0_* when there is low, but selective, conversion, then at high conversions %pe would be minimal. Whilst this is technically correct, it is not practically applicable to preparative photochemistry. Therefore, we propose the concept of *competitive yield*, which is defined as the yield of one photoproduct minus the yield of the other. We note that due to the complexity involved in the nomenclature around this topic, we have added a glossary to the Supporting Information that clarifies the definitions of key terms used in this manuscript. The evolution of the competitive yield over time is shown on the right side of Figure 1c. For *λ* < 430 nm, **A’** is formed rapidly, with a high degree of selectivity. However, since the concentration of **A** is significantly decreased during the reaction, the competing cleavage of **B** takes place more prominently after a certain point, and the competitive yield reverts towards 0%, i.e., an equal ratio of **A’** and **B’** produced. For the wavelength regime 430 < *λ* < 440 nm, the reverse trend is observed, where initially **B’** is formed, but as it depopulates more **A’** is generated later in the reaction (for timeframes longer than the 5 min showed).

Since the concept of competitive yield is only applicable to a binary mixture and only gives information on the relative excess of each species instead of the absolute amount produced, we also propose the concept of *reaction trajectories*. Here, each axis represents the concentration of the compound released in an irradiation experiment at a given wavelength – the trajectories are plotted as time‐dependent concentration evolutions in this space. Each trajectory starts at the origin and ends eventually at the end of the diagonal (100 µM/100 µM in our case), however each trajectory does so at its own rate.

The reaction trajectories of our system, up to a simulated irradiation time of 5 min, are visualised in Figure 1d. The colour of the trajectory matches the colour of the irradiating light. Ideal trajectories come as close to pure product release as possible. For 375 nm, after 10 s, **A’** is released almost completely, whereas with longer irradiation times the trajectory turns parallel to the *y*‐axis, indicating more release of **B’**. For optimal release of compound **B’**, the simulations suggest an irradiation wavelength of 445 nm for 5 min. A deviation of only 5 nm down to  440 nm results in more rapid cleavage of **B’**, but the selectivity of the reaction becomes significantly lower, with higher concentrations of **A’** also being liberated. Irradiating at the isosbestic point of the OD·*Φ*_λ_ plot (ca. 430 nm) will release both products at equal rates – after 1 min 50% of each will be cleaved. Figure 1d further demonstrates how small changes in the wavelength can cause dramatic differences in the outcome, reinforcing the importance of fine wavelength resolution of all techniques used to characterise a system when conducting precision photochemistry.

**Figure 2:**
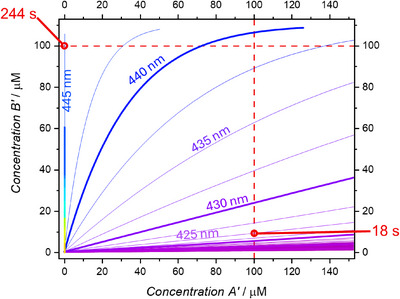
Reaction trajectories at different wavelengths for the cleavage of 500 µM of A and 110 µM of B. The trajectories are coloured using the colour that corresponds to the wavelength in the electromagnetic spectrum. The first wavelength is 315 nm and the final one is 590 nm with a 2.5 nm step between each simulation. 10 nm intervals are highlighted in bold and 100 µM concentrations are shown with red dashed lines.

With the four pillars (*ε_λ_*, *Φ*_λ_, *c*, and *t*) and a given choice of chromophores and environmental conditions (for example solvent and temperature), three degrees of experimental freedom remain: the choice of irradiation wavelength, the starting concentrations and the irradiation time. The importance of the starting concentrations is often underestimated – they do not have to be identical. The trajectory at 425 nm in Figure 1d, which was calculated for a 100 µM concentration of both **A** and **B**, can be used as an example where no real selectivity can be obtained. For example, irradiating at 425 nm for 5 min, liberates a 100 µM concentration of **A’** and 99.6 µM concentration of **B’** (Figure 1d). Figure 2 shows the results of a simulation starting from 500 µM of **A** and 110 µM of **B** and otherwise identical conditions to the simulation shown in Figure 1d. A desymmetrisation of the starting conditions stretches the release trajectories along the respective axes (Figure 2). Consequently, just 18 s of irradiation at 425 nm are sufficient to also liberate 103 µM of **A’** but now only 9.3 µM of **B’** are produced at the same time. 100 µM of **B’** can be released at 445 nm after 244 s without any release of **A’**. This shows how precise control of starting concentrations can compensate for less‐than‐ideal reaction quantum yields at a given wavelength. These aspects also need to be considered for systems that require only specific target concentrations to be achieved. Here, we assume both to be identical (100 µM–represented by dashed red lines in Figure 2). In the planning of such experiments, trajectories which cross these finish lines as closely to one of the axes as possible are preferred.

Although many traditional photochemical approaches consider factors such as absorbance, light intensity, sample concentration and more, the present work innovates an alternative approach that formulates the four‐dimensional matrix between time, concentration, wavelength‐dependent absorptivity and quantum yield, enabling researchers to predict reaction outcomes in complex systems with a lower barrier‐to‐entry. Our simulations visualise the importance of considering each of the four pillars of precision photochemistry to yield the optimal set of photoproducts without overshooting the ideal conditions. In fact, we propose that ideally every photochemical process should be subjected to analysis, as described in the example, to accurately determine its optimum operational window for a given application. Our analysis can be readily extended to other reaction types and is not limited to photo‐uncaging reactions.

## 2 Definition of Precision Photochemistry

Thus far, we have visualised the impact that the careful consideration of each of the four pillars of photochemistry can have on the reaction outcomes of a dual release photo‐uncaging system. As we demonstrated, the selection of specific wavelengths, concentrations, and irradiation times, when conducting photochemistry, enables light to be used not as a blunt energy source – as has been common for centuries^[^ ^22^ ^]^ – but as a tool with surgical precision that allows a chemist to achieve specific reaction outcomes depending on their application. By standardising the approaches used by researchers that apply photochemistry as a tool across the academic landscape, not only will their experiments progress with less opportunity for competing‐ or side‐reactivity, but reproducibility in the field of photochemistry will also be dramatically increased, a matter that is in critical need of improvement.^[^ ^23^, ^24^ ^]^

On the basis of the aforementioned points, there is a need for a unifying definition of the field of *Precision Photochemistry* – we propose the following:

*Precision photochemistry is the targeted cleavage, formation, and/or rearrangement of a quantifiable number of chemical bonds upon irradiation with photons of a known flux and given monochromatic wavelength(s)*.

In the remainder of this perspective, we will interrogate the literature to highlight examples that further show the importance of *Precision Photochemistry*. We will subsequently present key considerations that are recommended to all (synthetic) photochemists.

## 3 Precision Photochemistry in Action

Over the last decade, there have been numerous literature examples where researchers have used some of the principles of precision photochemistry to achieve specific goals. We propose that these areas will strongly benefit by viewing precision photochemistry through the lens of its four defining parameters *ε*_λ_, *Φ*_λ_, *t*, and *c* as demonstrated above.

### 3.1 Precision Photochemistry in Organic Chemistry

In synthetic organic chemistry, photochemistry is a powerful tool for controlling bond formation and cleavage pathways. By adjusting the wavelength of light, molar extinction coefficient, and photon flux, precise spatial and temporal control over molecular assembly can be achieved. For example, the ability to uncage a functional group in a molecule with a photocleavable‐protecting groups (PPG) is one molecular approach that has been extensively investigated for controlling chemical processes with light.^[^ ^25^ ^]^ By designing PPGs that are wavelength‐orthogonal and exhibit differences in molar extinction (*ε*_λ_) and reaction quantum yield (*Φ*_λ_) compared to other PPGs, a protecting group can be selectively removed in any order, even in the presence of other classes of PPG. Numerous groups have exploited this approach to achieve non‐invasive spatiotemporal control over the release of molecules of interest including biologically active compounds,^[^ ^26^, ^27^ ^]^ synthetic precursors,^[^ ^25^ ^]^ fluorescent probes,^[^ ^28^ ^]^ initiators of polymerisation reactions,^[^ ^4^, ^29^, ^30^ ^]^ and biomaterials engineering.^[^ ^31^ ^]^

A recent example leveraged photochemical action plots to develop wavelength‐dependent cleavage of a hydrogel network on‐demand with three different wavelengths (420, 365, and 325 nm).^[^ ^32^ ^]^ Furthermore, the photochemical efficiency and behaviour of cycloaddition reactions, including [2 + 2] cycloadditions, 1,3‐dipolar cycloaddition, and [4 + 2] cycloaddition have been significantly enhanced through systematic studies of their wavelength‐dependent reactivity.^[^ ^33^, ^34^, ^35^, ^36^ ^]^ For example, the [2 + 2] cycloaddition of acryl‐amidylpyrene, with its absorption maximum close to 370 nm, remains reactive at wavelengths exceeding 490 nm.^[^ ^37^ ^]^ This significant discovery would have been overlooked if more than just *ε*_λ_ had not been considered, thus highlighting the importance of considering all of the four pillars, even when conducting photochemistry with single‐chromophores.

By applying the illustrated concepts to *o*‐quinodimethane and 1,4‐diaryltetrazole motifs, a similarly red‐shifted reactivity in comparison with its extinction spectrum was revealed.^[^ ^35^, ^36^, ^37^, ^38^ ^]^ Furthermore, recent studies using photochemical action plots have elucidated the efficiency of *N‐*aryl azacycle ring contraction at wavelengths longer than those strongly absorbed by the substrate.^[^ ^39^ ^]^ Taken together, the consistently observed variation in *Φ*_λ_, highlights the need for a deeper understanding and characterisation of wavelength‐dependent reactivity to enable the rational design and optimisation of related photochemical transformations.^[^ ^5^ ^]^

### 3.2 Precision Photochemistry in Material Science

Due to the low energetic costs and high spatiotemporal control associated with photochemistry, light has also become a common stimulus for designing smart materials. The incorporation of photoswitches into a bulk material offers the unique ability to exploit nanoscopic conformational switching to impart macroscopic change on a material in response to irradiation.

For example, in liquid‐crystal elastomers (LCEs), soft materials can be programmed to exhibit specific photomechanics by incorporation of photoswitches such as molecular motors,^[^ ^40^ ^]^ or donor‐acceptor Stenhouse adducts (DASAs).^[^ ^41^ ^]^ In the latter case, Read de Alaniz and coworkers utilised the inverse photochromism of a DASA to create LCEs that bleach upon irradiation with lower intensity light, then actuate upon irradiation with higher intensity light. This is a direct demonstration of the optimisation of photon flux to achieve a precise photochemical outcome. The sequential bleaching of the DASAs, through the volume, in response to low‐intensity light means that as the irradiation progresses the light can penetrate further into the material, thus highlighting the importance of *t*, *c*, and *ε_λ_* in this field.

Azobenzene photoswitches have also been used to modulate the material properties of supramolecular networks through their ability to reversibly intercalate with cyclodextrin in one state but not the other.^[^ ^42^ ^]^ This fact has been exploited to induce swelling in nanogels that allows the controlled release of pharmaceutical cargos upon irradiation with light that triggers photoisomerisation.^[^ ^43^, ^44^ ^]^ These different photoswitches are able to be independently triggered in a wavelength orthogonal manner, as demonstrated by the team of Feringa.^[^ ^9^, ^10^, ^11^, ^12^, ^13^, ^14^, ^15^, ^16^, ^17^, ^18^, ^19^, ^20^, ^21^, ^22^, ^23^, ^24^, ^25^, ^26^, ^27^, ^28^, ^29^, ^30^, ^31^, ^32^, ^33^, ^34^, ^35^, ^36^, ^37^, ^38^, ^39^, ^40^, ^41^, ^42^, ^43^, ^44^, ^45^ ^]^ Upon incorporation into a material matrix, this would provide the opportunity to use precision photochemistry to modulate both the opacity and the physical material properties independently.

Photochemical decrosslinking has also been achieved by incorporation of *o*‐nitrobenzyl,^[^ ^46^ ^]^ dithioacetals,^[^ ^47^ ^]^ bimanes,^[^ ^48^ ^]^ and many other motifs in the crosslinking units of materials. The alternative case has also been demonstrated, where moieties such as anthracene are used to regenerate material properties after thermal degradation.^[^ ^49^, ^50^, ^51^ ^]^ These approaches allow the on‐demand modulation of physical properties such as swelling ratio, modulus, and hardness. The precise modulation of these important material properties is governed by the degree of conversion achieved in the photochemical reactions within the network. Additionally, photocuring of resins and coatings is highly dependent on the conversion of photochemical moieties (typically into radicals), and the kinetics at which they react. Thus, adopting an approach that carefully considers the impact of *ε_λ_*, *Φ_λ_*, *t*, and *c* on these molecular events, as well as factors such as penetration depth and scatter,^[^ ^20^ ^]^ will facilitate careful targeting of specific, desired material properties.

### 3.3 Precision Photochemistry in Additive Manufacturing

Perhaps the most common example of the use of photochemistry in the context of materials is in the field of additive manufacturing. Photochemical developments have enabled techniques such as stereolithography (SLA),^[^ ^52^ ^]^ continuous liquid interface production (CLIP),^[^ ^53^ ^]^ and multi‐colour laser printing^[^ ^54^ ^]^ to efficiently fabricate intricate and functional objects. Recent innovations leveraging distinct wavelengths of light have unlocked unprecedented opportunities in 3D printing. For instance, xolography and light‐sheet printing utilise synergistic photochemistry,^[^ ^55^, ^56^ ^]^ where simultaneous irradiation with two distinct wavelengths triggers reactions to occur – this has led to an enhancement in the printing speed that can be achieved. Conversely, stimulated emission depletion (STED)‐based nanoprinting employs antagonistic systems–separately activating opposing reactions with different wavelengths–to achieve resolution beyond the diffraction limit of light.^[^ ^12^ ^]^

Techniques such as grayscale printing,^[^ ^57^ ^]^ which modulate light intensities within a single photoresist, have enabled the fabrication of soft/hard or degradable/non‐degradable components.^[^ ^57^, ^58^ ^]^ More recently, Fors and colleagues have also showed that it is possible to modulate the composition and material properties of a network by simply altering the duration of irradiation, *t*.^[^ ^59^ ^]^ However, fully wavelength‐orthogonal 3D printing, where distinct reactions are independently triggered by different colours of light, is yet to be realised. Several groups have exploited semi‐orthogonal photochemistry to fabricate hard and soft sections within one object,^[^ ^60^, ^61^, ^62^, ^63^ ^]^ and recent advances by the Barner–Kowollik group demonstrate the potential of fully orthogonal photochemistry to produce materials and objects with integrated degradable/non‐degradable and soft/hard properties.^[^ ^58^, ^64^, ^65^ ^]^ To achieve such wavelength orthogonality, photochemical action plots of two chromophores, mapping wavelength‐dependent quantum yields, guided the precise wavelength selection.

Key parameters including molar extinction coefficients (*ε*_λ_), chromophore concentrations (*c*) and exposure times (*t*) must also be systematically studied to define an orthogonal printing window. Moving forward, developing photochemical reactions alongside advanced simulations considering the interaction of the four pillars, and real‐time, in‐situ characterisation will be critical to refine and optimise these processes. Such advancements will further expand the material and structural complexity achievable with precision photochemistry in 3D printing, unlocking new horizons for additive manufacturing.

### 3.4 Precision Photochemistry in Chemical Biology

Photochemistry has become an important tool in chemical biology for the triggering of biological activity, as seen in photopharmacology, and to make the invisible fluorescent; essential for biomedical imaging. There are two principal approaches for the regulation of biological activity by light: photolabile protecting groups that trigger biological processes irreversibly (referred to as photocages), and photoswitches that allow the reversible mediation of biological functions. Both of these methods have been applied to a wide range of biological targets.^[^ ^66^ ^]^ Wavelength‐resolved uncaging in the context of chemical biology was realised by the Heckel team in 2013 when they used four different photocages for oligonucleotides that could be sequentially cleaved, moving from low to high energy light.^[^ ^67^ ^]^ Later, the same team investigated two of the pillars of precision photochemistry, *c* and *t*, when they introduced the concept of chromatic selectivity – this enabled the near‐sequence‐independent orthogonal release of two chromophores by applying the aforementioned mathematical framework.^[^ ^17^ ^]^ They also demonstrated that, without knowledge of all the four pillars, reaction rates are able to be used to get an approximation of precision photochemistry.^[^ ^68^ ^]^

As mentioned previously, photoswitches operate between two metastable states that exist in ratios that are governed by wavelength‐dependent equilibria known as photostationary states.^[^ ^69^ ^]^ These states are dependent on the irradiation wavelength, *ε*_λ_, and *Φ*_λ_ – all of which should be known to inform the design of photochemical systems for use in chemical biology. In the case of nucleic acids, photochromic nucleosides have become an important tool for modulating the 3D structure of oligonucleotides, and for making reversibly fluorescent DNA labels.^[^ ^70^ ^]^ Photoswitching has also enabled the field of optogenetics whereby photochemical modulation of a protein structure either activates or deactivates a given function.^[^ ^71^ ^]^ This has become particularly critical for gaining control over neural activity.^[^ ^72^, ^73^, ^74^, ^75^ ^]^

The spatiotemporal control afforded by light that has been exploited by optogenetics can also be translated into precision control over pharmaceutical function. Activation of prodrugs with light has been a popular strategy for many years now. In particular, the photochemical uncaging of a cytotoxic compound from a non‐cytotoxic host has been investigated for application in cancer treatment.^[^ ^76^, ^77^, ^78^, ^79^ ^]^ High energy photons (X‐rays), that are used in radiotherapy, have even been utilised to simultaneously release chemotherapeutics by photochemical uncaging.^[^ ^80^ ^]^ Though prodrugs are advantageous when designing therapeutics that are intended to kill cells, having reversible control over the activation of a drug brings even further degrees of precision in *t*. This field, known as photopharmacology incorporates photoswitches that are biologically active in one state, and dormant in their other state.^[^ ^81^, ^82^, ^83^, ^84^ ^]^

Not only to be used as a therapeutic tool, but also as a diagnostic one, precision photochemistry is at the forefront of fluorescence labelling for biological applications. Photoligation reactions have become important for bioconjugation,^[^ ^85^, ^86^ ^]^ for example the photoinduced reaction of diaryltetrazoles where alkenes, first described by Huisgen in the late 1960s,^[^ ^87^ ^]^ were applied for conjugation of proteins,^[^ ^88^ ^]^ glycans,^[^ ^89^ ^]^ and nucleic acids.^[^ ^90^, ^91^ ^]^ Here, precision photochemistry is important for discovering excitation wavelengths that are suitable for live cell applications (typically in the redshifted region). Photochemical action plots of pyrene‐modified tetrazoles revealed their enhanced *Φ*_λ_ in the visible light range – redshifted when compared to the *ε*_λ_.^[^ ^38^ ^]^ Thus, less cytotoxic, red‐shifted light, that also has deeper penetration into the medium with less scatter can be used for the metabolic labelling of DNA in cells.^[^ ^92^ ^]^ The Wagenknecht team then showed that these reactions are able to operate with additional orthogonality to other bio‐orthogonal photoreactions using two geometrically altered reaction partners as reacting groups.^[^ ^93^ ^]^ The development of more efficient, type IV photoclick reactions has also enabled the photochemical synthesis of, for example, ^18^F‐positron emission tomography tracers – critical in the detection and treatment of diseases such as prostate cancer.^[^ ^94^, ^95^, ^96^ ^]^

### 3.5 Precision Photochemistry in Photocatalysis

Photoredox catalysis links the physical process of absorption to a desired reaction in organic chemistry.^[^ ^97^, ^98^, ^99^ ^]^ It overcomes the general problem that – for many systems – the energy content of visible light (and in the nearby UV‐A) range is significantly lower compared to the energy needed for direct excitation of a chemical bond. Instead, substrate radical ions are generated as reactive intermediates to gain selectivity. In general, both inorganic complexes, mainly [Ru(bpy)_3_]^2+^ and organic photoredox catalysts are excited at their respective absorption bands according to the parameters λ and *ε*_λ_. The reaction quantum yield *Φ*_λ_ is only rarely found in literature^[^ ^100^ ^]^ although it is an important parameter for photoredox catalysis: *Φ*_λ_ is not only dependent on *λ*, but also on the irradiation power of the light source if a radical chain reaction competes with the photocatalytic reaction.^[^ ^101^ ^]^

The concept of precision photochemistry is of growing importance for photoredox catalysis. In the initial pioneering work of MacMillan and colleagues,^[^ ^99^ ^]^ light was used and referred to as a “photon source”. In less than two decades, the field has grown and adopted more aspects of precision photochemistry – for instance, the activity and selectivity of transition metal catalysts can be switched by light if the catalyst bears a photoresponsive group.^[^ ^102^ ^]^ It has also been shown that it is possible to gain wavelength‐control over photoredox catalysis by using a system where bond activation is controlled through the wavelength‐selective regulation of redox potentials.^[^ ^103^ ^]^ Alternatively, it is also possible to change the photocatalytic selectivity by altering the intensity (or photon flux) of the irradiation. Wenger's team demonstrated that the selectivity of a water‐soluble Iridium photocatalyst can be switched by changing from a one‐photon to a two‐photon absorption process.^[^ ^104^ ^]^ Multi‐photon activation in general has attracted attention in the context of photoredox catalysis due to the low propensity for photo‐induced side reactions to occur, as is common with high‐energy UV light.^[^ ^105^ ^]^ Instead of multiple photons being absorbed in a single event, it is also possible to have sequential absorption events, effectively charging the redox system to enable photocatalysis–this approach is known as consecutive photoinduced electron transfer (conPET).^[^ ^106^ ^]^ The Wagenknecht team showed recently that the photocatalytic activity of *N*‐arylphenothiazines can be tuned in a synergistic way by the irradiation at two distinct wavelengths.^[^ ^107^ ^]^ This methodology enables the photocatalytic introduction of pentafluorosulfanyl groups into organic compounds by activating sulfur hexafluoride.^[^ ^108^, ^109^ ^]^

## 4 Practical Implementation of Precision Photochemistry

It would be remiss of us to emphasise the critical importance of undertaking *Precision Photochemistry* without providing the required guidance in how to do so. When such experiments are undertaken, there are several key parameters which must be reported: i) the photon dose, ii) the experimental geometry, iii) the characterisation method and conditions, and iv) the wavelength‐dependent reaction quantum yield. In what follows, we will outline these key factors in detail.

### 4.1 Light Sources

In the early days of photochemistry, the light source typically employed was unfiltered, unfocussed solar radiation.^[^ ^22^, ^23^, ^24^, ^25^, ^26^, ^27^, ^28^, ^29^, ^30^, ^31^, ^32^, ^33^, ^34^, ^35^, ^36^, ^37^, ^38^, ^39^, ^40^, ^41^, ^42^, ^43^, ^44^, ^45^, ^46^, ^47^, ^48^, ^49^, ^50^, ^51^, ^52^, ^53^, ^54^, ^55^, ^56^, ^57^, ^58^, ^59^, ^60^, ^61^, ^62^, ^63^, ^64^, ^65^, ^66^, ^67^, ^68^, ^69^, ^70^, ^71^, ^72^, ^73^, ^74^, ^75^, ^76^, ^77^, ^78^, ^79^, ^80^, ^81^, ^82^, ^83^, ^84^, ^85^, ^86^, ^87^, ^88^, ^89^, ^90^, ^91^, ^92^, ^93^, ^94^, ^95^, ^96^, ^97^, ^98^, ^99^, ^100^, ^101^, ^102^, ^103^, ^104^, ^105^, ^106^, ^107^, ^108^, ^109^, ^110^ ^]^ Being a blackbody emitter, the solar spectrum is extremely broad, meaning that the wavelength of the irradiation was rarely considered a factor – the light was seen merely as a source of energy. Some control over the wavelength can be gained by using diffractive optics to separate the light, as demonstrated by Trommsdorff in 1834 when he reported the wavelength‐dependent decomposition of α‐santonin.^[^ ^111^ ^]^

**Figure 3:**
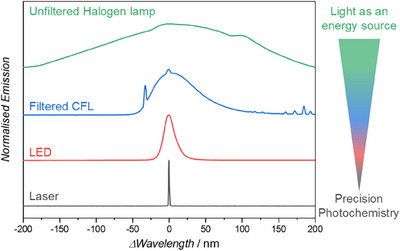
The emission spectra of commonly employed light sources normalised to their maximum emission wavelength.

Artificial light sources such as halogen lamps, mercury lamps, or compact fluorescent lamps (CFLs) often have very broad emission spectra (Figure 3, green), owing to the blackbody spectrum, numerous atomic emission lines or broad fluorescence spectra, respectively. In industrial photochemistry, broad emission poses an engineering challenge since byproducts are often formed, antagonistic reaction channels limit yields, photostationary states are reached, or the photoproducts degrade thus preventing high yields from being achieved. Additionally, the low energetic efficiency of these light sources makes them, environmentally speaking, less favourable.^[^ ^112^ ^]^ The broad spectral emission can be somewhat negated by using filters that select certain wavelength regions to either be filtered out (cut‐off filters), or to be allowed through (band‐pass filters) (Figure 3, blue).^[^ ^113^, ^114^ ^]^ Although filtering light can be beneficial, the energetic efficiency of the light source is extremely low since many photons are lost in the process.^[^ ^114^ ^]^

More recent technological advances in engineered light sources have enabled more careful selection of the irradiation wavelength and increased the efficiency of the irradiation process. Light emitting diodes (LEDs, Figure 3, red) are now readily available at low cost, with high power output, and with a wide range of available wavelengths. Furthermore, the invention of lasers in the 1960′s has enabled monochromatic sources of light, which can be tuned across broad wavelength range with the help of optical parametric oscillators. Both of these advances have been awarded Nobel Prizes accordingly.

When conducting precision photochemistry, it is critical that the spectral emission of the light source is sufficiently narrow, so that it does not trigger any competing photochemical processes. For example, if the starting material is activated at one wavelength (*λ*_1_), and the photoproduct can be degraded at another wavelength (*λ*_2_), the spectral emission of the light source should be sufficiently narrow that its maximum emission can be centred at *λ*_1_, yet for it to be negligible at *λ*_2_. In the context of reporting light sources in precision photochemical studies, it is essential to report the emission characteristics of the employed light source in both spectral distribution and intensity. These parameters can be measured directly with the aid of a spectrophotometer. Alternatively, the combination of a spectrometer and energy meter can be used to measure the spectrum and total emitted intensity. The intensity at each wavelength can then be calculated by integrating over the spectral distribution. Note that not all energy sensors are created equal. Photodiodes are ideal for low intensity LED or continuous wave laser light, while thermopile sensors are optimised for high intensity light, such as that from pulsed lasers. The use of a sensor for the wrong application will result in inaccurate readings.

For high precision experiments – including recording photochemical action plots – lasers also provide highly coherent light that is not available from other light sources, such as LEDs, thus allowing access to certain photochemical reactions or processes that are dependent on incident light phase (such as non‐linear processes).^[^ ^115^ ^]^ Pulsed laser systems are also able to deliver light at high peak intensities that are not typically achievable from other light sources, enabling better access to multiphoton processes, excited state absorption events or absorption of short‐lived reaction intermediates.

### 4.2 Experimental Geometry

Equally important as where the photons originate from is their trajectory through space. Key to a successful photoreaction is optimising the intensity or number of photons into the reaction mixture, so that they are absorbed and subsequently induce chemical change. In the determination of the quantum yield of some chemical actinometers, changes in the experimental setup–and thus in the eventual number of photons truly absorbed–has resulted in a 34% discrepancy in their reported quantum yields in literature.^[^ ^116^ ^]^ Examples such as these highlight the need for precision in both the undertaking and reporting of photochemical reactions.

Variations in both light source and optical components, as well as their placement, are integral in obtaining precise photon delivery to a sample for quantum yield measurements. For example, LED emission is highly divergent, and hence without any collimation optics, the intensity of the light drops off at a rate proportional to the distance squared. Condenser lenses can be utilised to counteract this divergence and optimise the amount of light that is delivered to the sample. The power of light incident upon the sample can be calculated simply as the product of the known light intensity and the product area.

Once the irradiation conditions are optimised, it is necessary to quantify the photon flux into the sample. The photon flux, defined as the number of photons incident on the sample per unit time, can be calculated with knowledge of the power and wavelength of the light source (as outlined in the Supplementary Information). The other key factor is the transmission of light through the reactor vessel, which must be experimentally validated, and not just calculated from manufacturer specifications in order to account for any variation in fabrication. In addition, variations in light emission over time must be accounted for, with LED intensity and wavelength both varying as the temperature of the device changes. Thus, the total number of photons incident on the sample can be calculated as we have outlined in the Supplementary Information.^[^ ^5^ ^]^ In line with the simulations presented at the beginning of the current perspective, photons can then be considered as a stochiometric reagent in photochemistry.

In addition to being important when considering the analytical aspects of photochemistry, the reaction geometry is integral in preparative photochemistry. For example, the penetration of light through a high concentration reaction mixture can mean that photons are only able to be absorbed near the surface. Thus, mechanical stirring is important when conducting batch‐reactions where photoproducts or intermediates can absorb or scatter the incident light. On the other hand, stirrers induce significant scattering and therefore undermine the ability to accurately quantify the photon flux. The walls of the reactor can also often cause significant scatter or lensing effects that make the light field non‐uniform, and results in the loss of photons. Hence, industrial batch‐reactors often immerse the light source directly in the reactor, meaning that all photons that are emitted come into contact with the reaction mixture.

An alternative way to mitigate penetration depth issue is to conduct photochemistry in flow. Photoflow chemistry has the benefit that low diameter tubing can be used, meaning that photons can fully penetrate into the reaction vessel and irradiate the whole reaction volume. It also has the advantage of scalability, since it is a continuous process,^[^ ^117^ ^]^ making photoflow chemistry one the most promising candidates for the industrialisation of photochemistry.^[^ ^118^, ^119^ ^]^ Recent developments in reactor geometry have also included using vortex reactors that generate thin films on reactor walls,^[^ ^120^, ^121^, ^122^ ^]^ allowing full penetration of the solution in a high‐shear environment that has the potential to enable photothermal processes to proceed rapidly, in flow.

### 4.3 Characterisation of Photoproducts

Where possible, it is ideal to perform the analysis of the photochemical conversion in situ to avoid any inaccuracies due to ambient light, degradation or reversion. Where this is not possible, care should be taken to make sure that the reaction is terminated immediately (e.g., for oxygen‐sensitive reactions, by exposing the sample to air) and the sample is protected from stray light until characterisation of the products can be performed. Any delays between exposure and characterisation should be clearly noted.

Common characterisation methods include ^1^H NMR, UV–vis absorption spectroscopy, mass spectrometry, react IR, and gravimetry. Each of these techniques has unique advantages (e.g., ^1^H NMR provides structural information, yet is very unsensitive), so careful selection of the correct method that allows quantification is key to reporting accurate data. Ideally, multiple characterisation methods that present corroborating kinetic data should be presented. After characterisation, the conversion should be reported, including the uncertainty, encompassing all measurement and procedural factors.

### 4.4 Quantum Yield Determination

Determining the wavelength‐dependent quantum yield of photophysical and photochemical processes has become considerably simpler using modern analytical equipment, including via photochemical action plots in a wavelength resolved fashion. For example, determining fluorescence quantum yields by measuring the relative photoluminescence compared to a standard has become a common practice in many laboratories globally through a variety of methods.^[^ ^123^, ^124^, ^125^, ^126^ ^]^ The determination of other processes, in particular photochemical transformations, can present more of an experimental challenge. The quantum yield (*Φ*_λ_) of a process is defined as the number of distinct events which occur per photon absorbed by the system – thus, a fluorescence quantum yield of one implies that every time a photon is absorbed by the molecule a subsequent photon is emitted through fluorescence.^[^ ^127^ ^]^ Therefore, it is imperative to determine the number of photons that are absorbed by a system to determine the quantum yield.

In the case of fluorescence, the quantum yield is often determined by finding the gradient of the slope of the emission‐absorption graph and relating it to a known reference standard. Typically, low concentrations (maintaining an optical density OD < 0.1) are used for these experiments to avoid inner filter effects. In photochemical reactions however, it is common that concentrations much higher than these are required to ensure that the reaction partners meet in solution whilst in an excited state, as well a new species being generated consistently which may or may not absorb the incident light. Therefore, it is not sufficient to simply compare photons absorbed (*N_abs_*) and reaction events. Instead, photon flux gradients across both the sample volume and time, as well as the evolution of the chemical species, must be taken into account. This methodology has been commonly employed when conducting photochemical action plots, and in our investigation of various organic chromophores.^[^ ^8^, ^15^, ^128^, ^129^, ^130^, ^131^ ^]^

An alternative approach to determining the photon flux, enabling the calculation of the reaction quantum yield, is to use photochemical actinometers. These reference chemicals have a known quantum yield, so their conversion can be used to calculate how many photons made it into the reactor.^[^ ^132^ ^]^ However, this can be challenging to accurately determine if a wavelength‐dependent quantum yield exists due to a lack of wavelength‐dependent standards in the literature. For this, versatile actinometers that absorb broad sections of the electromagnetic spectrum are needed, such as those developed by Heckel and team.^[^ ^18^ ^]^ Robust methodologies have been established around using photochemical actinometers to determine the quantum yields of photochemical reactions.^[^ ^133^ ^]^ Further practical guidance on calculating quantum yield values has been provided in the Supplementary Information, as well as in a variety of literature references.^[^ ^8^, ^17^, ^128^, ^130^, ^133^, ^134^, ^135^, ^136^ ^]^

### 4.5 Expanding the Scope of the Four Pillars

On occasion, *ε*_λ_, *Φ*_λ_, or both may not be suitable for a certain application. For example, should the molar extinction be too high at the wavelength that you wish to irradiate, the penetration depth may be too low to enable meaningful curing in volumetric additive manufacturing techniques, or in chemical biology the *Φ*_λ_ may be low in the red‐shifted, non‐cytotoxic region of the electromagnetic spectrum. Photochemists have developed several methods to mitigate this, for example via photosensitisation.

A photosensitiser is a molecule that absorbs the light and subsequently, by some photophysical mechanism, transfers the energy to a reaction partner to enable photochemistry to take place.^[^ ^137^ ^]^ Often, these sensitisers work by having efficient intersystem crossing quantum yields that then transfer their energy to a reaction partner, decaying back to their ground state in the process. This approach has become somewhat commonplace in synthetic organic photochemistry, as it decreases reaction times and often allows for the red‐shifting of the irradiation wavelength – thus minimising potential side reactions.^[^ ^138^ ^]^ Another use is to design multicomponent photoreactive systems, for example the Page group has recently developed a semi‐orthogonal photoresin that functions by activation of a photosensitiser with red‐shifted light (only triggering one curing process), then activation of multiple curing processes simultaneously when irradiating with higher energy light – without the photosensitiser, the activation wavelength of the first mechanism would overlap with the activation of the other.^[^ ^60^ ^]^ Quantum dots, advantageous due to their tuneable band gap, have also been shown to be used as efficient light harvesters and photosensitisers for various reaction classes.^[^ ^139^ ^]^

When used in the presence of dissolved oxygen (^3^O_2_ in its ground state), triplet sensitisers are able to undergo triplet–triplet annihilation that regenerates the ground state sensitiser and highly reactive singlet oxygen (^1^O_2_). Generating singlet oxygen has found applications in organic synthesis,^[^ ^140^, ^141^ ^]^ photodynamic therapy,^[^ ^142^, ^143^ ^]^ antimicrobials,^[^ ^144^ ^]^ and even in photochemical laundry detergents.^[^ ^145^ ^]^

The same triplet–triplet annihilation process can be utilised in to generate one higher energy photon from multiple lower energy photons.^[^ ^146^ ^]^ By confining the multiple components needed for up‐conversion, either by entrapment in a molecular container,^[^ ^147^ ^]^ or encapsulation in micelles,^[^ ^148^, ^149^ ^]^ efficient up‐conversion can be achieved whilst maintaining low absolute *c*. This approach negates the issues that arise when high energy light is needed, but does not have the ability to penetrate deep enough into the medium, for example in volumetric additive manufacturing.^[^ ^148^ ^]^

Finally, multiphoton processes can be used to access different excited states, or to trigger reactions with enhanced spatial resolution within a medium. Multiphoton absorption has found many applications in additive manufacturing where it is used to go beyond the resolution limit possible in a standard printer,^[^ ^150^, ^151^ ^]^ photocatalysis,^[^ ^105^ ^]^ and photodynamic therapies.^[^ ^143^ ^]^ Careful design of the chromophore system can also give rise to complex excited state landscapes where multiple absorption events can occur in sequence, known as (1 + 1)‐photon absorption.^[^ ^152^ ^]^ These multiphoton processes are highly dependent on the light intensity, and thus a tightly focussed, coherent laser is essential to their success.

## 5 Conclusions

We have demonstrated the critical importance of considering each of the four pillars of precision photochemistry when conducting chemistry with photons: the molar extinction (*ε*_λ_), the wavelength‐dependent reaction quantum yield (*Φ*_λ_), the duration of the irradiation (*t*), and the concentration of the chromophores in solution (*c*). Initially, we showed via simulations of the photochemical reactivity of a photo‐uncaging system that each of these four parameters must be known and accounted for in order to realise the full potential of photochemistry. We then presented a unifying definition for this growing field of precision photochemistry and put forward our thoughts on several research fields that can directly benefit from implementation of the methods we outlined, namely small molecule synthetic organic photochemistry, materials science, chemical biology, additive manufacturing, and photocatalysis. Whilst not exhaustive, the breadth of fields in the above list shows the potential for the precision photochemistry approach to benefit many researchers working across different disciplines.

Finally, we explored some key considerations that should be adhered to by all researchers wishing to get the most out of every photon when conducting precision photochemistry – we hope that this article will function as a useful reference for interdisciplinary researchers beginning their photochemistry journey. By carefully unpacking the role that each photon has on a photochemical process, we believe that many processes can be made more efficient, or strongly improved within the framework of precision photochemistry.

## Supporting Information

The authors have cited additional references within the Supporting Information.

## Conflict of Interests

The authors declare no conflict of interest.

## Supporting information



Supporting Information

## Data Availability

The data that support the findings of this study are available from the corresponding author upon reasonable request
