# Supplementary material for: Precision Photochemistry: Every Photon Counts
Source: Angew Chem Int Ed Engl. 2025 Aug 4;64(35):e202502651. doi: 10.1002/anie.202502651 (PMC12377443; doi:10.1002/anie.202502651)
Supplement: Supplementary file 1 — Supporting Information [file ANIE-64-e202502651-s001.pdf]

Precision Photochemistry: Every Photon Counts

Supplementary Information

Methods for the determination of the number of photons incident on the sample

Photon flux determination from the output of a monochromatic light source

Initially, the power incident on the sample (P) must be determined. This can be simply calculated when the intensity of the light source on the sample (I) and the area of the sample exposed to light (a) are known:

$$P = I \times a.$$

This, in turn, can be used to determine the photon flux, q_p, incident upon the sample:

$$q_p = \frac{P\lambda\,[T(\lambda)/100]}{hc},$$

where $T(\lambda)$ is the wavelength dependent transmission through the sample vessel in %, h is Planck's constant and c is the speed of light.

This can then be turned into the total number of photons delivered to the sample, N_p, by multiplying by the exposure time of the experiment (t):

$$N_p = q_p \times t = \frac{P\lambda t\,[T(\lambda)/100]}{hc}.$$

Photon flux determination using an actinometer

The use of actinometers for the determination of photon flux has been well described elsewhere in the literature,[1-4] and these methods will not be reiterated here.

Methods for the determination of quantum yields

Reaction quantum yield determination tries to solve the equation:

$$\Phi(\lambda) = \frac{N_m \cdot \rho(\lambda)}{N_a},$$

where N_m is the number of molecules being irradiated, N_a is the number of photons absorbed, $\rho(\lambda)$ is the wavelength dependent conversion of the reaction, and $\Phi(\lambda)$ is the wavelength dependent reaction quantum yield. In a crude approximation, when the change in absorbance between the starting material and the photoproduct is

negligible, and the conversion from starting material to photoproduct is also kept low, the following approximation can be made:

$$\Phi(\lambda) = \frac{c \cdot V \cdot N_A \cdot \rho(\lambda)}{N_p \cdot (1 - 10^{-\varepsilon(\lambda)cl})},$$

where c is the concentration of the solution, V is the volume of the solution, N_A is Avogadro's Number, $\varepsilon(\lambda)$ is the wavelength dependent molar extinction coefficient and l is the path length of the irradiation.

When a significant difference in the absorbance over time is observed, the equation must be integrated over the time of the irradiation:

$$\Phi(\lambda) = \int_0^t \frac{c(t) \cdot V \cdot N_A \cdot \rho(\lambda, t)}{N_p(t) \cdot (1 - 10^{-A(\lambda,t)})} dt,$$

where $A(\lambda, t)$ is the wavelength dependent and time dependent absorbance of the sample, accounting for the wavelength dependent molar absorptivity, time dependent concentration, and potentially a time dependent path length. In some situations, more complex methods such as simulating for each pulse of a pulsed laser can be used,[5-6] but they will not be discussed here. Gescheidt has also presented a useful tutorial on how to calculate the reaction quantum yield using an actinometer.[1]

Mathematical basis for the reaction outcome simulations

All simulations presented in Figure 1 and Figure 2 were calculated using the mathematical framework that has been laid out by Heckel and co-workers.[2] Using molar extinction coefficients (ε) of the starting materials and photoproducts, and wavelength dependent quantum yields ($\Phi(\lambda)$) as a inputs, the differential equations presented below are used to calculate the reaction outcomes at each wavelength for a binary mixture of A and B.

$$\frac{dA}{dt} = -\frac{q_p}{V}(1 - 10^{-l(\varepsilon_{A'}A' + \varepsilon_A A + \varepsilon_B B + \varepsilon_{B'}B')}) \frac{\varepsilon_A A}{\varepsilon_{A'}A' + \varepsilon_A A + \varepsilon_B B + \varepsilon_{B'}B'} \Phi(\lambda)_{AA'}$$

$$\frac{dB}{dt} = -\frac{q_p}{V}\left(1 - 10^{-l(\varepsilon_{A'}A' + \varepsilon_A A + \varepsilon_B B + \varepsilon_{B'}B')}\right) \frac{\varepsilon_B B}{\varepsilon_{A'}A' + \varepsilon_A A + \varepsilon_B B + \varepsilon_{B'}B'} \Phi(\lambda)_{BB'}$$

incident photons

percentage of absorbed photons

part absorbed by A or B, respectively

quantum yield for the respective reaction

For an in-depth discussion of these equations, see [2]. Note that A or B denotes the concentration of the respective species. These differential equations were solved using custom-made software that we seek to make publicly available in the future.

Glossary of Terms

Given the complexity of the nomenclature in the field of *Precision Photochemistry*, the key technical terms used are defined here. Where possible, definitions are taken from the IUPAC recommendations.[7]

**Absorbance, *A***	The logarithm to the base 10 of the ratio of the spectral radiant power of incident, essentially monochromatic, radiation to the radiant power of transmitted radiation. Simply, a measure of the absorbing power of a solution/substance. Absorbance measurements assume there is no scattering of light, and absorbance is dimensionless. $$\boldsymbol{A = \log\frac{I_0}{I} = -\log T}$$ $\boldsymbol{A}$ is absorbance, $\boldsymbol{I_0}$ is the initial intensity of the light source, $\boldsymbol{I}$ is the intensity of the light transmitted by the sample, $\boldsymbol{T}$ is the transmittance.
**Actinometer**	A chemical system or physical device which determines the number of photons in a beam integrally or per unit time. This name is commonly applied to devices used in the ultraviolet and visible wavelength ranges.
**Action Plot**	Or Photochemical Action Plot is a modern-day adaptation of action spectroscopy, where a monochromatic untenable ns-pulsed laser is used to interrogate photochemical systems in a wavelength-by-wavelength manner, yielding the wavelength dependent quantum yield Φ_λ.
**Action Spectrum**	A plot of a relative biological or chemical photoresponse per number of incident photons, against wavelength or energy of radiation under the same radiantpower of light. This form of presentation is frequently used in the studies of biological or solid-state systems, where the nature of the absorbing species is unknown.
**Band-pass filter**	An optical device which permits the transmission of radiation within a specified wavelength range and does not permit transmission of radiation at higher or lower wavelengths. It can be an interference filter.
**Bathochromic- or red-shift**	Shift of a spectral band to lower frequencies (longer wavelengths) owing to the influence of substitution or a change in environment (e.g. solvent).
**Bleaching**	In photochemistry, bleaching is the loss of absorption or emission intensity.
**Coherent radiation**	A source emits coherent radiation when all elementary waves emitted have a phase difference constant in space and time.
**Cut-off filter**	An optical device which only permits the transmission of radiation of wavelengths that are longer than or shorter than a specified wavelength. Usually, the term refers to devices which transmit radiation of wavelengths longer than the specified wavelength

**Extinction**	The sum of the effects of absorbance and scattering - this is what is measured in a UV/vis spectrometer.
**Hypsochromic- or blue-shift**	Shift of a spectral band to higher frequencies (shorter wavelengths) owing to the influence of substitution or a change in environment (e.g. solvent).
**Inner Filter Effect**	This term is used in two different ways. In an emission experiment, it refers to an apparent decrease in emission quantum yield and/or distortion of band shape as a result of reabsorption of emitted radiation. During a light irradiation experiment, absorption of incident radiation by a species other than the intended primary absorber is also described as an inner filter effect.
**Intensity**	Traditional term for photon flux, fluence rate, irradiance or radiant power (radiant flux). In terms of an object exposed to radiation, the term should now be used only for qualitative descriptions.
**Irradiation time**	The duration of an irradiation regardless of the nature of the irradiation.
**Laser**	A source of ultraviolet, visible, or infrared radiation which produces light - amplification by stimulated emission of radiation from which the acronym is derived. The light emitted is coherent except for superradiance emission.
**Molar Extinction Coefficient or Molar Absorbtion Coefficient ε_λ**	Absorbance or extinction divided by the absorption pathlength (l) and the concentration (c). Usually given in units of $M^{-1}cm^{-1}$. $$A = \varepsilon c l$$
**Non-linear optical effects**	An effect brought about by electromagnetic radiation the magnitude of which is not proportional to the irradiance. Non-linear optical effects of importance to photochemists are harmonic frequency generation, lasers, Raman shifting, upconversion, and others.
**Optical Density (OD)**	Synonymous with Absorbance
**Penetration Depth**	The length at which the radiant power of a source is 10% of its initial power at the source.
**Photochemical Reaction**	This term is generally used to describe a chemical reaction caused by absorption of ultraviolet, visible, or infrared radiation. There are many ground state reactions which have photochemical counterparts. Among these are photoadditions, photocycloadditions, photoeliminations, photoenolizations, photo-Fries rearrangements, photoisomerisations, photooxidations, photoreductions, photosubstitutions, etc.
**Photochemistry**	The branch of chemistry concerned with the chemical effects of light (far UV to IR).
**Photon**	The quantum of electromagnetic energy at a given frequency. The energy of a photon is given by: $$E = h\nu = \frac{hc}{\lambda}$$ E is energy of the photon, h is Planck's Constant, ν is the frequency of the light, c is the speed of light, λ is the wavelength of the light.
**Photon dose**	The total number of photons deposited into a sample at the completion of an irradiation.

**Photon Equivalents**	The molar ratio of the number of photons used to a given reagent. Similarly to equivalents in organic chemistry, where the photon is considered a reagent
**Photon flux**	The photon number of photons incident on an object per unit time.
**Photosensiti-sation**	The process by which a photochemical or photophysical alteration occurs in one molecular entity as a result of initial absorption of radiation by another molecular entity called a photosensitiser.
**Photostationary state**	A steady state reached by a reacting chemical system when light has been absorbed by at least one of the components. At this state the rates of formation and disappearance are equal for each of the transient molecular entities formed.
**Precision Photochemistry**	The targeted cleavage, formation, and/or rearrangement of a quantifiable number of chemical bonds upon irradiation with photons of a known flux and given monochromatic wavelength(s).
**Quantum yield** $\boldsymbol{\Phi}$	The number of defined events which occur per photon absorbed by the system.
**Transmittance** $\boldsymbol{T}$	The ratio of the transmitted intensity of light to that incident on a sample
**Wavelength** $\boldsymbol{\lambda}$	The distance, measured along the line of propagation, between two corresponding points on adjacent waves. The wavelength depends on the medium in which the wave propagates.

References

[1] E. Stadler, A. Eibel, D. Fast, H. Freissmuth, C. Holly, M. Wiech, N. Moszner, G. Gescheidt, *Photochem. Photobiol. Sci.* **2018**, *17*, 660-669.

[2] M. Reinfelds, V. Hermanns, T. Halbritter, J. Wachtveitl, M. Braun, T. Slanina, A. Heckel, *ChemPhotoChem* **2019**, *3*, 441-449.

[3] J. Rabani, H. Mamane, D. Pousty, J. R. Bolton, *Photochem. Photobiol.* **2021**, *97*, 873-902.

[4] L. de Brito Anton, A. I. Silverman, J. N. Apell, *Environ. Sci.: Processes Impacts* **2024**, *26*, 1052-1063.

[5] F. Feist, L. L. Rodrigues, S. L. Walden, T. W. Krappitz, T. R. Dargaville, T. Weil, A. S. Goldmann, J. P. Blinco, C. Barner-Kowollik, *J. Am. Chem. Soc.* **2020**, *142*, 7744-7748.

[6] P. W. Kamm, L. L. Rodrigues, S. L. Walden, J. P. Blinco, A.-N. Unterreiner, C. Barner-Kowollik, *Chem. Sci.* **2022**, *13*, 531-535.

[7] J. W. Verhoeven, *Pure Appl. Chem.* **1996**, *68*, 2223-2286.
